# New Sign That a Child Has Been Born Alive

**Published:** 1851-11

**Authors:** 


*New Sign that a Child has been Born Alive.* — Dr. Virchow has announced that the presence of uric acid in the kidney, which may be detected with the naked eye, is conclusive of a child having been born alive. His conclusions are —

1. That uric acid deposit is never found in children born dead, or who have died within forty-eight hours after birth.

2. That the deposit did not occur before forty-eight hours after birth.

3. That it is not generally found later than the twentieth day after birth.

